# Schizophrenia Genetics: Building the Foundations of the Future

**DOI:** 10.1093/schbul/sbu162

**Published:** 2014-11-13

**Authors:** Katherine E. Tansey, Michael J. Owen, Michael C. O’Donovan

**Affiliations:** MRC Centre for Neuropsychiatric Genetics and Genomics, Institute of Psychological Medicine and Clinical Neurosciences, School of Medicine, Cardiff University, Cardiff, UK

In recent years, our understanding of the genetic architecture of schizophrenia, a phrase which denotes the numbers of risk variants, their frequencies and effect sizes, has been transformed. This has come about through advances in technology that have allowed almost the entire human genome to be simultaneously interrogated for the presence of disease-associated genetic variation and allows this to be performed in sample sizes powered for a realistic possibility of success. Another development has been the emergence of international consortia willing to share raw data and their coalescence into super-consortia to achieve sample sizes and bodies of clinical and analytic expertise that was unimaginable a decade ago. These innovations have driven the emergence of statistically robust and replicable genetic findings in schizophrenia, and a rapid escalation in the number of those findings over the last 5 years.

The latest example comes from the Schizophrenia Working Group of the Psychiatric Genomics Consortium (PGC-SCZ) which, at the time of publication, included contributions from around 37 000 individuals with schizophrenia, 302 investigators, 35 countries, and 4 continents.^1^ In their recent paper, published in *Nature* in July 2014, the PGC-SCZ group report 128 statistically independent genetic associations, implicating a minimum of 108 conservatively defined schizophrenia-associated genetic loci.^1^

Of the identified loci, 83 have not been previously robustly supported as playing a role in schizophrenia, but it is also important to note the findings are consistent with previous literature; 25 loci that had previously been reported as associated with schizophrenia in large samples were again supported in this much larger analysis, confirming that the use of large samples and stringent statistical cut-offs results in reproducible findings. The availability of so many robustly supported findings offers immense opportunities for investigating and advancing our understanding of etiology.

## Large Numbers of Alleles Across the Frequency Spectrum

While each individual variant themselves have a small effect size (less than 1.4), it has been estimated that common variation as a whole accounts for around a third to half of the genetic variance in schizophrenia^2,3^ though this may turn out to be an underestimate when heterogeneity and interaction effects are taken into account. Nevertheless, rare variants also play a role. This has been well established for around 20 years with respect to small deletions and duplications known as copy number variants (CNVs)^4,5^ but systematic surveys for other forms of rare genetic variation have had to await the development of high capacity next generation sequencing technology. These studies, particularly the larger ones,^6,7^ have been commented on in a recent review in the journal^8^ and will not be considered in detail here. However, like the early genome-wide association studies (GWASs),^2,9^ rather than striking evidence for individual susceptibility genes, the recent sequencing studies provided evidence that many loci contribute to risk and for enrichment of rare mutations in certain gene sets. This strongly suggests that, as we have seen for GWAS, better powered studies will implicate specific genes.^10,11^

The evidence that risk variants for schizophrenia occur across the range of allele frequencies is compelling. As noted above, the contribution to risk of schizophrenia arising from the aggregate effect of common variants is not trivial, but even a single common variant can have a similar effect on the variance in the population as rare variants with larger effects simply because they occur much more frequently in the population.^12^ This suggests that in order to understand genetic risk mechanisms we will need to explore all parts of the allele frequency spectrum. Moreover, it seems likely that advantages may accrue from a combined approach to gene identification as the PGC-SCZ study pointed to an overlap between genes in schizophrenia GWAS regions and those with de novo mutations. This suggests that a combination of the superior power of GWAS (via sample size) and the superior precision of sequencing with respect to gene identity might on the one hand be usefully harnessed to identify likely causal genes within GWAS-associated loci and on the other to enhance the power of sequencing by providing targets with enhanced prior probability.

## Genetic Associations to Biological Mechanisms

The identity of individual associations, and patterns of enrichment of various types of associations and disease-linked mutations are beginning to shed light on areas of biology that are likely relevant to schizophrenia, although we stress detailed mechanistic conclusions will require other types of research, and that the associations at each locus have not yet been firmly linked to specific genes. Nevertheless, it is notable that within the schizophrenia-associated loci are multiple genes involved in synaptic function and plasticity, particularly genes involved in glutamatergic neurotransmission (*GRM3*, *GRIN2A*, *GRIA1*, and *SLC38A7*) and neuronal calcium signaling (*CACNA1C*, *CACNB2*, *CAMKK2*, *CACNA1I*, *NRGN*, and *RIMS1*). *DRD2*, which a priori is possibly the strongest of all conceivable candidate genes for schizophrenia based on function, is also associated with the disorder.

Given that most of the common variant associations do not appear to result from DNA changes that affect protein sequence, it is presumed, with some evidence,^13^ that they exert their effects through influencing gene expression. Investigating this hypothesis further, the PGC-SCZ group sought to determine if schizophrenia-associated common variants are concentrated in regulatory elements marked as activating gene expression in particular tissues or cell lines. Importantly, though largely as predicted, associations were enriched in these regulatory elements in various brain tissues and in genes showing high expression in neurons/interneurons. A much more novel and potentially important finding was they were also enriched in these regulatory elements in the immune system, particularly B-lymphocyte cell lineages. This finding is intriguing as it provides some genetic, and therefore etiological, support for the general hypothesis that immune dysregulation plays a role in the development of schizophrenia.^14^ However, we need to move beyond general enrichment analyses to identify specific causal variants in specific regulatory elements, understand which genes/proteins are affected by those regulatory elements, and show how genetic variation directly affects immune but not neuronal function, before the immune hypothesis of schizophrenia can be considered to be genetically confirmed.

The limited network analyses conducted by the PGC-SCZ did not identify any generically annotated biological pathways that were enriched for associations. This may reflect the restricted analyses presented, and a more thorough evaluation of the data is underway. But it may also reflect either high polygenicity and/or limitations in the quality and availability of data upon which to base these bioinformatic-driven analyses. Not only are the functions of many proteins unknown, but even less well documented are the elements that regulate the expression and processing of protein isoforms in specific cellular, developmental, and physiological contexts especially in the brain. Proteomic studies in particular lack the comprehensive scale of transcriptomic studies and are currently limited to targeted approaches. Interestingly, in PGC-SCZ, support was found for one of the most consistently implicated gene sets in schizophrenia, a set comprised of brain-expressed genes that interact with the Fragile X mental retardation (FMRP) protein.^3,6,7,15^ While the biological implications of this are not yet understood, it is likely that improving knowledge of the protein interactome and of the constituent members of sets of proteins involved in brain function will improve our ability to move from patterns of association to pathogenesis. This is likely to require more extensive experimental validation, and iterative refinement, of bioinformatics tools. For example, experimental studies reveal little overlap between genes predicted to be targets of microRNA-137, whose encoding gene lies within a schizophrenia-associated locus, and those whose expression is affected by knockdown or overexpression of the microRNA^16^ demonstrating that experimental validation of bioinformatics predictions is essential.

Targets of FMRP are not the only consistently implicated gene set in schizophrenia. Both GWAS^1,3^ and exome sequencing^7^ points to the involvement of multiple calcium channels and multiple genes involved in calcium signaling, a process also implicated in bipolar disorder.^17,18^ Proteins affiliated with the *N*-methyl-d-aspartate receptor and the activity-regulated cytoskeleton-associated protein have been implicated by CNVs analysis^19^ and by exome sequencing,^6,7,20^ while as noted above, *GRIN2A* and multiple proteins related to glutamatergic signaling are associated with the disorder in the recent GWAS.^1^ These gene sets plausibly converge at the functional level of synaptic plasticity and remodeling,^6,21^ although this hypothesis requires testing through mechanistic experimental studies.

The complexity and inaccessibility of human brain tissue has made it challenging to understand basic disease mechanisms and to translate genetic findings into biology. Discussed more fully in a recent review,^8^ a synthesis of gene discovery with recent advancements in stem cell technology and genome engineering mean that an exciting avenue of research has been opened for psychiatry. This could be either through patient-derived induced pluripotent stem cells^22^ or through manipulations of cell lines by emerging gene engineering technologies.^23,24^ Further discussion of these developing areas is outside the scope of the current article but have been discussed elsewhere.^25–33^

## Pleiotropy

Pleiotropy occurs when one gene or genetic variant contributes to multiple phenotypes, a phenomenon fast becoming a characteristic of identified genetic risk factors for neuropsychiatric disorders. There is already evidence for extensive sharing of common genetic risk variants between schizophrenia, bipolar disorder major depressive disorder, and attention deficit hyperactivity disorder (ADHD)^17,34,35^ though the evidence for ADHD is somewhat less consistent than that for the other phenotypes. The high genetic correlation is not explained by diagnostic misclassification,^36^ and instead points to considerable genetic pleiotropy in terms of these categorically defined diagnoses. The same is true for rare genetic risk factors for schizophrenia. It has been clear for several years that the same CNVs that confer risk for schizophrenia also do so for neurodevelopmental disorders including autism spectrum disorders (ASD), intellectual disability, ADHD, and epilepsy.^4,5,37,38^ Given most of these CNVs affect multiple genes, it could conceivably be argued that different genes were involved in each phenotype, but a recent analysis of point mutations supports the view that sharing of risk occurs at the level of genes and types of mutation.^6^ Moreover, the PGC-SCZ study found overlap between the genes in schizophrenia GWAS regions and those with de novo mutations in intellectual disability and ASD providing further support for overlapping genetic risk, and presumably pathophysiology.

The extent of pleiotropy may be surprising, but it is consistent with other disciplines which generally show high rates of comorbidity and lack of specificity in disease associations. Pleiotropy can also facilitate understanding of disease mechanisms by identifying novel intermediate phenotypes on the causal chain. For example, many risk factors for type 2 diabetes are pleiotropic for body mass and are likely to mediate their effects on the former through the latter. Of course in type 2 diabetes, there was a strong hypothesized link with body mass, and exploiting pleiotropy is not going to be quite so simple in psychiatry. Rather, finding pleiotropic links may require deep, and largely speculative, mass phenotyping. Deep phenotyping refers to the collection of large amounts of information on individuals beyond categorical diagnostic status, eg, cognitive and neuroimaging data, or given recent findings (see above) immune function. It is early days with respect to this type of work, but promising results have already been demonstrated for the first GWAS-identified schizophrenia risk gene, *ZNF804A*,^9^ which have been associated with cognition, clinical subdimensions, and brain phenotypes.^39–42^ Small samples limit the robustness of the conclusions that have emerged so far from this sort of research.

Exploiting pleiotropy is likely to require very large cohorts, although much smaller samples ascertained for the same relatively high penetrance mutation, eg, a CNV, are also likely to be highly informative. The considerable genetic overlap between disorders also suggests it will be important that much of this work should be undertaken across current diagnostic boundaries (and also include unaffected individuals given we all carry large numbers of common risk alleles) in order to characterize the impact of genetic variants.

## From Genetics to Treatment

Antipsychotics are only partly effective for the positive symptoms of schizophrenia and do little to alleviate the negative symptoms or cognitive deficits. No novel class of drug for schizophrenia has emerged since the 1960s,^43^ presumably due to the limited understanding and insight into the molecular underpinnings of schizophrenia. It is therefore some cause for optimism that the recent PGC-SCZ publication implicates genetic variants at the locus that contains *DRD2*, the gene that encodes the D2 dopamine receptor, the target of all known effective antipsychotics. The association of genetic variants at this gene can be seen as a “proof of principle” finding in reverse, supporting the hypothesis that pharmacological manipulation of proteins highlighted by common variants can have substantial therapeutic effects regardless of the fact that the genetic effect size on risk is meagre.^44^ It also suggests that other loci implicated by GWAS harbor the potential to guide effective drug discovery for schizophrenia, although we need better knowledge of the actual risk variants, and the proximal and distal functional consequences of those variants in the pathway to disease.

Furthermore, each gene implicated in the development of schizophrenia does not work independently. If the gene itself is an unsuitable drug target, there is the potential for manipulation via interacting proteins or other members participating in the same biological pathway. Therefore, it is important to fully characterize these risk genes and the cellular process, pathways, and phenotypes which they regulate. Translating just one associated locus into an effective treatment for schizophrenia would amply justify the contribution made by tens of thousands of patients with the disorder that have made the recent genetic advances possible, and the investments of effort and resource by researchers and funding bodies, governmental and charitable.

## Conclusions

The recent PGC-SCZ publication is a landmark in the process of identifying genetic risk variants for schizophrenia, but it is not one that indicates the end of the journey. As sample sizes and power increases, and with the additional detail provided by sequencing, discovery is likely to accelerate. However, even now, the genetic findings provide the basis for a wealth of research, from molecular and cellular investigations through to defining novel clinical classifications. It is too early to know the implications for new treatments, although some of the associations may immediately inspire potential therapeutic targets. What would seem inevitable is that if as yet undiscovered treatments for the disorder are possible, and it seems unlikely that the only effective treatment has already been discovered, the opportunities for better understanding of pathogenesis that flow from the genetic data must surely accelerate their discovery. For this to happen, scientists of many disciplines must move away from the comfort of some of the “old favourites,” for which the evidence is much less secure and devote their energies and intellect in pursuit of the new findings that are well grounded in evidence. Looking beyond schizophrenia, the findings definitively demonstrate the power of genetics can be harnessed for psychiatric phenotypes despite their presumed high heterogeneity, the absence of tests to validate diagnosis, and uncertain biological validity.

## Funding

The work at Cardiff University was funded by Medical Research Council (MRC) Centre (G0800509) and Program Grants (G0801418); European Community’s Seventh Framework Programme (HEALTH-F2-2010-241909, Project EU-GEI).
